# Clinical Validation of Plasma p‐217tau in Neurological Diseases

**DOI:** 10.1002/acn3.70359

**Published:** 2026-03-09

**Authors:** Takeshi Kawarabayashi, Takumi Nakamura, Ryoma Takahashi, Tetsuya Ueda, Seiji Kinoshita, Chikage Uchida, Takashi Sugawara, Kentaro Hashimoto, Kunihiko Ishizawa, Masakuni Amari, Hiroo Kasahara, Yoshio Ikeda, Masamitsu Takatama, Mikio Shoji

**Affiliations:** ^1^ Department of Neurology Dementia Research Center, Geriatrics Research Institute and Hospital Maebashi Japan; ^2^ Department of Neurology Gunma University Graduate School of Medicine Maebashi Japan; ^3^ Department of Social Medicine Hirosaki University Graduate School of Medicine Hirosaki Japan; ^4^ Analytical Research Center Mediford Corporation Tokyo Japan

**Keywords:** Alzheimer's disease, amyotrophic lateral sclerosis, idiopathic normal pressure hydrocephalus, plasma p‐tau, p‐tau217

## Abstract

**Objective:**

Plasma p‐217tau is a minimally invasive but specific biomarker for diagnosing Alzheimer's disease (AD). However, its disease specificity remains to be clinically evaluated. We validated the reliability of the p‐217tau biomarker in 12 other neurological diseases.

**Methods:**

Plasma p‐217tau levels were measured in 298 participants, consisting of 81 AD patients, 204 patients with 12 other neurological diseases, and 13 healthy and cognitively unimpaired controls (HCU), using an assay system from Meso Scale Diagnostics. Cerebrospinal fluid (CSF) tau and Aß levels were simultaneously evaluated in AD, amyotrophic lateral sclerosis (ALS), and idiopathic normal pressure hydrocephalus (iNPH).

**Results:**

Plasma p‐217tau levels increased in AD with the clinical stage, but also in ALS and iNPH, leading to them having decreased sensitivity and specificity for diagnosing AD. No increases in plasma p‐217tau levels were seen in possible tauopathies or synucleinopathies. CSF and plasma p‐217tau levels were strongly correlated in AD, but not in ALS. The plasma p‐217tau/CSF p‐217tau ratio was inversely higher in ALS than in AD. Active and chronic denervation potentials were associated with plasma p‐217tau levels. In iNPH, plasma p‐217tau was associated with cognitive dysfunction, but not with gait disturbance or urinary incontinence. CSF p‐181tau, total tau, and Aß1‐40 levels and the Aß1‐40/1–42 ratio were reduced in iNPH.

**Interpretation:**

ALS and iNPH are two major pitfalls for the clinical application of plasma p‐217tau as a biomarker of AD. Lower motor neuron injury in ALS and cognitive dysfunction in iNPH were both found to be associated with elevated plasma p‐217tau levels.

## 1 Introduction

In the Alzheimer's disease (AD) continuum, amyloid‐ß (Aß) deposition starts 2 decades before the onset of symptomatic cognitive decline, and it is followed by the accumulation of phosphorylated tau. After a long incubation period, neurofibrillary tangles and neurodegeneration emerge. Cognitive decline progresses to clinical dementia, which lasts for more than 10 years. The signatures of these pathological and clinical processes are traced using biomarkers that are present in cerebrospinal fluid (CSF) or plasma as well as via positron emission tomography (PET) of Aß amyloid and tau amyloid deposition [1, 2, 3]. Among these biomarkers, tau phosphorylated at threonine 217 (p‐217tau) emerges in CSF and plasma early from Braak stage III, and its level continuously increases to the severe Braak stage VI over 4 decades [4, 5, 6]. Increased CSF and plasma p‐217tau levels are closely associated with Aß amyloid deposits [7, 8, 9, 10]. The high accuracy of plasma p‐217tau for identifying AD pathology has been demonstrated without PET‐ or CSF‐based confirmatory testing [11, 12]. Large cohort studies further confirmed that plasma tests are of similar or superior utility to clinical CSF biomarkers [13, 14, 15]. Based on these findings, the plasma p‐217tau level has recently been proposed as a core 1 biomarker for diagnosis, staging, and prognosis in the National Institute on Aging and Alzheimer's Association's (NIA‐AA) revised criteria for the diagnosis and staging of AD [16].

Plasma p‐217tau was shown to be highly accurate for differentiating AD and AD‐related syndromes from other neurological diseases, such as Parkinson's disease (PD), multiple system atrophy (MSA), frontotemporal dementia (FTD), progressive supranuclear palsy (PSP), corticobasal syndrome (CBS), vascular dementia, and dementia with Lewy bodies (DLB) [17, 18, 19]. However, increased plasma p‐217tau levels have recently been reported in amyotrophic lateral sclerosis (ALS) [20], chronic kidney disease [21], cardiovascular disease factors [22], and newborns [23]. For this reason, it is necessary to validate neurological diseases and physical conditions which increase plasma p‐217tau levels, to enable the clinical use of the plasma p‐217tau level as a specific and sensitive biomarker of AD pathology, and to rule out other pathological conditions. Here, we validated the utility of the plasma p‐217tau level as a biomarker of AD by assessing plasma p‐217tau levels in 12 other neurological diseases.

## 2 Methods

### 2.1 Subjects

The subjects of the present study were participants in the Biomarkers Initiative by Geriatrics Institute and Gunma University of Neurodegenerative Diseases (BIGUN) [24, 25, 26]. This cohort was studied to elucidate the longitudinal natural courses of biomarkers in neurological diseases in collaboration with Gunma University and the Geriatrics Research Institute and Hospital in Japan. The total number of participants in the BIGUN cohort was 546 (261 males and 285 females). The subjects' mean age was 65 ± 15 years. In the current study, we selected consecutively enrolled participants with complete data sets. A total of 298 plasma samples, consisting of samples from 81 AD patients, 204 patients with 12 other common neurological diseases, and 13 healthy and cognitively unimpaired control subjects (HCU), were examined (Table 1). All participants provided written informed consent. This study was approved by the ethics committee of Geriatrics Research Institute and Hospital (numbers 69 and 78), Gunma University (number IRB2021‐029), and Hirosaki University (number 2017‐112). The subjects were diagnosed based on the criteria listed in the following references: AD dementia, mild cognitive impairment due to AD, and AD cognitively unimpaired [27], cerebral amyloid angiopathy (CAA) [28], and cortical cerebellar atrophy (CCA) based on adult‐onset slow progressive ataxia with a negative family history [29].

**TABLE 1:** Patients' profiles, diseases, cognitive function, and plasma p‐217tau levels.

Disease	*N*	F/M	Age	MMSE	p‐217tau (pg/mL)
AD	81	50/31	69 ± 11	21.6 ± 6.1	^§^22.98 ± 14.59
ADD	40	24/17	68 ± 12	18.1 ± 6.3	27.05 ± 14.68
ADMCI	38	24/13	70 ± 9	24.6 ± 3.5	19.70 ± 13.71
ADCU	3	2/1	51 ± 22	29.3 ± 1.2	10.13 ± 7.10
CAA	11	5/6	69 ± 13	20.6 ± 8.2	8.13 ± 8.30
DLB	2	2/0	68, 70	13, 29	7.95, 1.75
FTD	5	1/4	66 ± 7	23.6 ± 2.7	2.89 ± 1.96
CBS	11	5/6	72 ± 6	22.2 ± 5.4	3.92 ± 1.97
PSP	18	6/12	73 ± 7	22.4 ± 5.2	4.54 ± 2.39
PD	32	22/10	67 ± 10	27.2 ± 2.8	3.15 ± 1.35
MSA	21	9/12	67 ± 9	26.2 ± 2.7	3.49 ± 1.74
CCA	5	4/1	56 ± 9	25.0 ± 5.7	3.48 ± 1.62
ALS	40	16/24	68 ± 8	25.9 ± 4.6	*6.82 ± 4.25
iNPH	40	21/19	78 ± 7	20.6 ± 6.5	*8.07 ± 7.19
MS	15	14/1	37 ± 14	n.e.	2.36 ± 0.95
NMOSD	4	3/1	55 ± 24	n.e.	5.15 ± 5.72
HCU	13	3/10	57 ± 16	29.3 ± 1.9	2.80 ± 1.14
Total	298	161/137	67 ± 13	22.9 ± 6.1	10.01 ± 11.68

*Note:* ^§^ *p* < 0.0001, *p < 0.05.

Abbreviations: ADCU, AD cognitively unimpaired; AD, Alzheimer's disease; ADD, AD dementia; ADMCI, mild cognitive impairment due to AD; ALS, amyotrophic lateral sclerosis; CAA, cerebral amyloid angiopathy; CBS, corticobasal syndrome; CCA, cortical cerebellar atrophy; DLB, dementia with Lewy bodies; FTD, frontotemporal dementia; HCU, healthy and cognitively unimpaired controls; iNPH, idiopathic normal pressure hydrocephalus; MS, multiple sclerosis; MSA, multiple system atrophy; ne, not examined; NMOSD, neuromyelitis optica spectrum disorders; NS, not significant; PD, Parkinson's disease; PSP, progressive supranuclear palsy.

The 40 ALS patients were diagnosed with the Gold Coast criteria [30]. Six had bulbar‐onset, and 34 had limb‐onset. Their clinical phenotypes consisted of 23 cases of the classic phenotype, 10 cases of the flail arms phenotype, 6 cases of the progressive bulbar palsy (PBP) phenotype, and an unclassified case. Clinical data relating to the following factors were collected: age at onset, time from onset to blood drawing, lower motor neuron (LMN) signs (muscle atrophy and hypotonia, or fasciculation), upper motor neuron (UMN) signs (hyperreflexia, spasticity, or pathological reflexes), the Medical Research Council (MRC) sum score [25], the UMN score [31], the ALS Functional Rating Scale‐Revised (ALSFRS‐R) score [32], the ALSFRS‐R progression rate, the partial pressures of carbon dioxide (PaCO_2_) and oxygen (PaO_2_) in arterial blood, and the percentage vital capacity (%VC). The presence/absence of active and chronic denervation were examined by electromyography (EMG) in the bulbar, cervical, thoracic, and lumbosacral spinal regions [33].

Idiopathic normal pressure hydrocephalus (iNPH) was defined according to the Guidelines for Management of Idiopathic Normal Pressure Hydrocephalus (Third Edition) [34]. All iNPH patients had neuroimaging features of narrowing of the sulci and subarachnoid space over the high‐convexity/midline surface (DESH). The iNPH patients underwent tap tests, and patients with high CSF p‐181tau levels (> 50 pg/mL) were excluded, as it was considered that their iNPH was a complication of AD. Gait disturbance was assessed with the timed up & go test (TUG) before and after (1 week later) the tap test. The TUG was carried out twice at each timepoint (TUG1 and TUG2).

Data regarding numbers of subjects, sex, age, Mini‐Mental State Examination (MMSE) scores, and plasma p‐217tau levels for all diseases are shown in Table 1. Compared with the HCU, the sex distributions of the AD, PD, CCA, iNPH, and neuromyelitis optica spectrum disorders (NMOSD) patients were different. The age distributions of the PSP and iNPH groups differed significantly from that of the HCU.

### 2.2 Assay of Plasma p‐217tau and Other CSF Biomarkers

Plasma p‐217tau levels were measured using an electrochemiluminescence assay from Meso Scale Diagnostics LLC, Rockville, MD (S‐PLEX; Cat. No.: K151APFS) [35]. Plates were read using a MESO QuickPlex SQ 120 instrument. The intra‐ and inter‐assay coefficients of variation were 7.7% and 2.0%. The range of detection of the assay was from 3.506 to 3590 pg/mL. CSF t‐tau, p‐181tau, and p‐217tau levels were measured using ELISA assays that we developed [24]. CSF Aß1‐40 and Aß1‐42 were measured with an enzyme immunoassay using the human amyloid (1–40) ELISA kit Wako II and the Human/Rat Amyloid (1–42) ELISA Kit Wako High‐Sensitive (Wako, Pure Chemical Industries Ltd., Osaka, Japan), respectively. The results are shown in Table 2. Since some patients did not agree to these examinations, the sample numbers for the various measurements differ.

**TABLE 2:** CSF biomarker levels in AD, ALS, and iNPH.

CSF biomarker (pg/mL)	AD	ALS	iNPH
p‐217tau	185.4 ± 97.8 *n* = 19	36.7 ± 37.1 *n* = 29	ne
p‐181tau	76.5 ± 31.2 *n* = 43	54.4 ± 37.3 *n* = 29	23.9 ± 17.8 *n* = 40
t‐tau	710.9 ± 292.0 *n* = 43	653.3 ± 256.9 *n* = 29	178.2 ± 96.3 *n* = 40
Aß1‐42	546.0 ± 271.3 *n* = 62	ne	630.1 ± 294.5 *n* = 40
Aß1‐40	9244 ± 3758 *n* = 62	ne	5731 ± 2416 *n* = 40
Aß1‐40/Aß1‐42 ratio	18.87 ± 7.89 *n* = 62	ne	9.508 ± 2.48 *n* = 40

*Note:* The mean ± standard deviation CSF p‐217tau, p‐181tau, t‐tau, Aß1‐42, Aß1‐40, Aß1‐40/Aß1‐42 levels in AD, ALS, and iNPH are shown. The number of subjects examined is described below the measured levels.

Abbreviation: ne, not examined.

### 2.3 Statistical Analyses

Since normality tests (the D'Agostino & Pearson, Anderson‐Daring, Shapiro–Wilk, and Kolmogorov–Smirnov tests) and normal QQ plot analysis showed that the data were not normally distributed, the Kruskal–Wallis test for nonparametric data was used. Dunn's multiple comparisons test was adopted as a post hoc test. The results of the statistical analyses are shown as mean ± standard deviation values in Tables 1 and 2. Area under the receiver operator characteristic curve (ROC) analysis was carried out to assess the diagnostic utility of the plasma p‐217tau level for discriminating between the AD patients and the HCU, between AD and the other neurological diseases, and between AD and ALS + iNPH. The optimal cutoff value was selected based on the likelihood ratio (sensitivity/[1 – specificity]) [36]. The Mann–Whitney test and Fisher's exact test were used for comparisons between two groups. Spearman's correlation coefficient was adopted for correlation analyses. All tests were two‐tailed and performed with GraphPad Prism, version 10 (GraphPad Software, San Diego, CA), and the significance level was set at 5%.

## 3 Results

### 3.1 Levels of p‐217tau in AD and Other Neurological Disease

**FIGURE 1:**
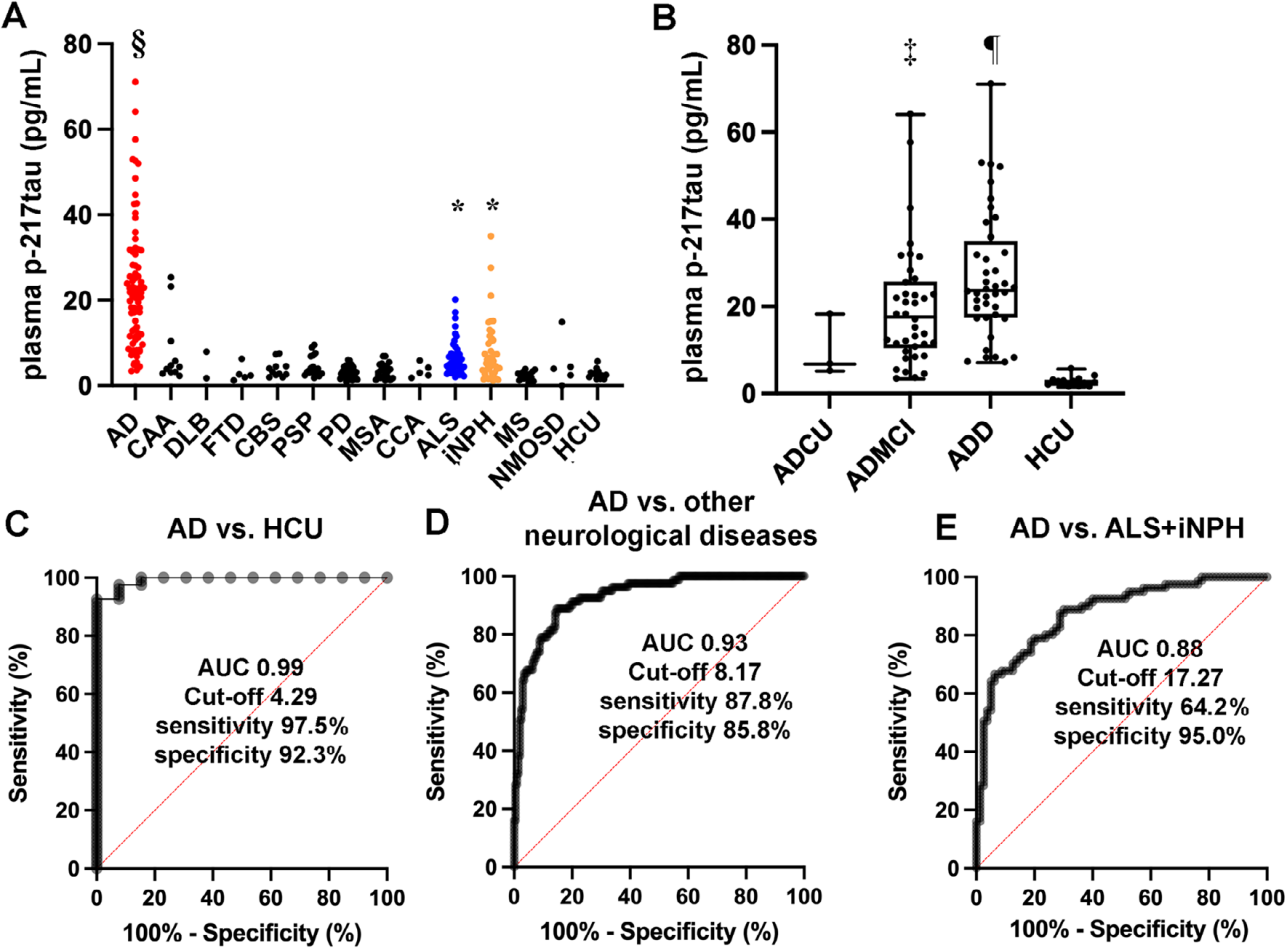
Plasma p‐217tau levels in various neurological diseases and ROC analysis. (A) Plasma p‐217tau levels in various neurological diseases; Plasma p‐217tau levels were significantly higher in the AD (§*p* < 0.0001), ALS (*p < 0.05), and iNPH patients (*p < 0.05) than in the HCU. (B) Plasma p‐217tau levels of ADCU, ADMCI (‡*p* < 0.001), and ADD patients (¶*p* < 0.01) compared with those of the HCU; (C) ROC analysis of the utility of the plasma p‐217tau level for differentiating AD patients from the HCU; The AUC was 0.99, the sensitivity was 97.5%, and the specificity was 92.3% at a cutoff value of 4.29 pg/mL. (D) ROC analysis of the utility of the plasma p‐217tau level for differentiating between AD patients and patients with other neurological diseases. The AUC was 0.93, the sensitivity was 87.8%, and the specificity was 85.8% at a cutoff value of 8.17 pg/mL. (E) ROC analysis of the utility of the plasma p‐217tau level for differentiating between AD patients and ALS and iNPH patients; The AUC was 0.88, the sensitivity was 64.2%, and the specificity was 95.0% at a cutoff value of 17.27 pg/mL.

The AD patients exhibited significantly higher plasma p‐217tau levels (22.98 ± 14.59 pg/mL) than the HCU (2.80 ± 1.14 pg/mL) (*p* < 0.0001, Table 1, Figure 1A, red dots). Plasma p‐217tau levels increased with clinical stage progression in AD (Figure 1B). Among the other neurological diseases, plasma p‐217tau levels were significantly higher in the patients with ALS (6.82 ± 4.25 pg/mL, blue dots) or iNPH (8.07 ± 7.19 pg/mL, yellow dots) than in the HCU (*p* < 0.05, Table 1, Figure 1A). The plasma p‐217tau levels of the ALS patients were 30% of those of the AD patients and 2.4‐fold higher than those of the HCU. The plasma p‐217tau levels of the iNPH patients were 35% of those of the AD patients and 2.9‐fold higher than those of the HCU. The plasma p‐217tau levels of the CAA patients were higher than those of the HCU; however, the difference was not significant. No increase in p‐217tau levels was seen in possible tauopathies, including FTD, CBS, and PSP, or synucleinopathies, including DLB, PD, and MSA.

### 3.2 ROC Analysis of Biomarker Utility for Diagnosing AD

In an assessment of the utility of the plasma p‐217tau level for differentiating between the AD patients and HCU, the area under the ROC (AUC) was 0.99. The optimal cut‐off value and associated sensitivity and specificity of the plasma p‐217tau level for differentiating the AD patients from the HCU were 4.29 pg/mL, 97.5%, and 92.3%, respectively (Figure 1C). Analyses of the utility of the plasma p‐217tau level for differentiating between AD and other neurological diseases showed that the associated AUC value was 0.93. The optimal cutoff value and associated sensitivity and specificity for differentiating AD from other neurological diseases were 8.17 pg/mL, 87.8%, and 85.8%, respectively (Figure 1D). The utility of the plasma p‐217tau level for differentiating AD from ALS and iNPH was decreased (AUC: 0.88, cutoff value: 17.27 pg/mL, sensitivity: 64.2%, and specificity: 95.0%) (Figure 1E).

### 3.3 Plasma and CSF p‐217tau Levels in ALS

**FIGURE 2:**
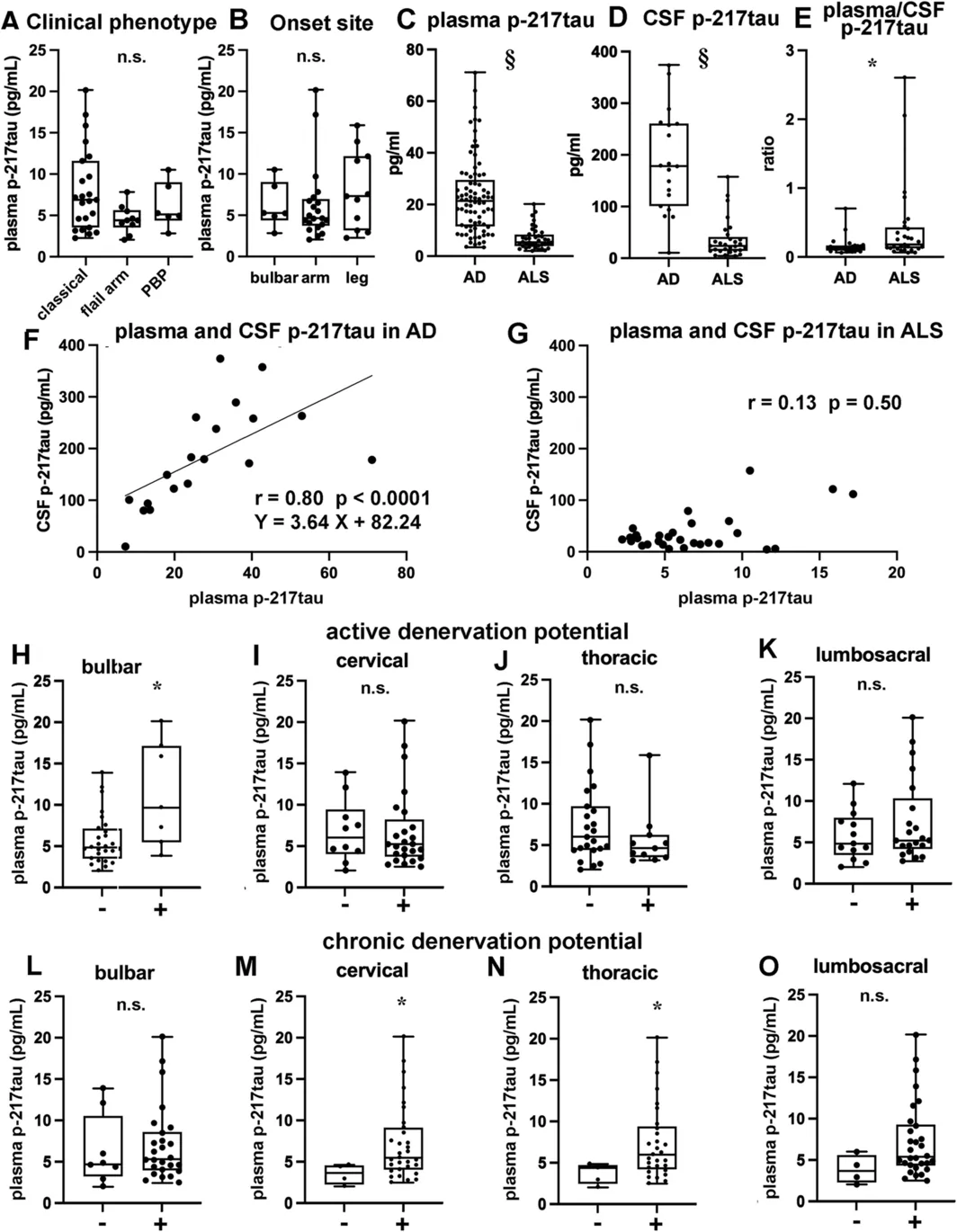
Associations between plasma p‐217tau and the clinical findings of ALS. The clinical phenotype (classical: 8.01 ± 5.01 pg/mL, *n* = 23; flail arm: 4.53 ± 1.67 pg/mL, *n* = 10; and PBP: 6.16 ± 2.81 pg/mL, *n* = 6; *p* = 0.12; A) and affected sites at onset (bulbar: 6.21 ± 2.74, *n* = 6; arms: 6.28 ± 4.55 pg/mL, *n* = 21; legs: 8.02 ± 4.71 pg/mL, *n* = 11; *p* = 0.46); (B) did not affect the plasma p‐217tau level. The mean plasma p‐217tau level of the ALS patients (7.19 ± 4.83 pg/mL) was 31% of that of the AD patients (22.98 ± 14.59 pg/mL); §*p* < 0.0001, (C). The mean CSF p‐217tau level of the ALS patients (36.7 ± 37.1 pg/mL) was 20% of that of the AD patients (185.4 ± 97.8 pg/mL), §*p* < 0.0001; (D). The mean plasma p‐217tau/CSF p‐217tau ratio was significantly higher in ALS (0.41 ± 0.58) than in AD (0.16 ± 0.13; *p < 0.05); (E). Plasma p‐217tau levels were strongly correlated with CSF p‐217tau levels in AD (*r* = 0.80), §*p* < 0.0001; (F); however, they were not correlated in ALS (*r* = 0.13), *p* = 0.50; (G). The plasma p‐217tau levels of the patients that did or did not exhibit active denervation potentials in the bulbar (negative: 5.79 ± 3.03 pg/mL, positive: 11.37 ± 6.34 pg/mL), *p < 0.05; (H), cervical (negative: 6.82 ± 3.86 pg/mL, positive: 6.82 ± 4.72 pg/mL), *p* = 0.77; (I), thoracic (negative: 7.54 ± 4.75 pg/mL, positive: 5.67 ± 3.62 pg/mL), *p* = 0.24; (J), or lumbosacral (negative: 5.87 ± 3.01 pg/mL, positive: 7.60 ± 5.10 pg/mL), *p* = 0.42; (K) region or chronic denervation potentials in the bulbar (negative: 6.37 ± 4.31 pg/mL, positive: 7.11 ± 4.56 pg/mL), *p* = 0.57; (L), cervical (negative: 3.51 ± 1.23 pg/mL, positive: 7.25 ± 4.53 pg/mL), *p < 0.05; (M), thoracic (negative: 3.78 ± 1.23 pg/mL, positive: 7.48 ± 4.58 pg/mL), *p < 0.05; (N), or lumbosacral (negative: 3.85 ± 1.73 pg/mL, positive: 7.35 ± 4.55 pg/mL, *p* = 0.06); (O) region; +, positive; −, negative; n.s., not significant.

In ALS, the mean CSF p‐217tau, p‐181tau, and t‐tau levels were 36.7 ± 37.1 pg/mL, 54.4 ± 37.3 pg/mL, and 653.3 ± 256.9 pg/mL, respectively (Table 2). The plasma p‐217tau levels of the ALS patients were 30% of those of the AD patients, and their CSF p‐217tau levels were 20% of those of the AD patients (Figure 2C,D). The plasma p‐217tau/CSF p‐217tau ratio was significantly higher in ALS (0.41 ± 0.58) than in AD (0.16 ± 0.13; *p* < 0.05; Figure 2E). Plasma p‐217tau levels were strongly correlated with CSF p‐217tau levels in AD (*r* = 0.80, *p* < 0.0001; Figure 2F); however, no such correlation was seen in ALS (*r* = 0.13, *p* = 0.50; Table 3, Figure 2G). No correlations were observed between plasma p‐217tau and CSF p‐181tau levels (*r* = 0.34, *p* = 0.07), or between plasma p‐217tau and CSF *t*‐tau levels (*r* = 0.03, *p* = 0.88) in ALS (Table 3). Thus, the increase in plasma p‐217tau levels seen in ALS was smaller than that observed in AD and was not associated with CSF p‐217tau levels.

**TABLE 3:** Correlation study: plasma p‐217tau and CSF biomarkers and clinical factors in ALS.

Subject	Correlation coefficient (*r*)	*p*
CSF p‐217tau	0.13	0.50
CSF p‐181tau	0.34	0.07
CSF t‐tau	0.03	0.88
Age at onset	0.25	0.12
Time since onset	0.16	0.32
MRC score	−0.21	0.20
UMN score	0.003	0.99
ALSFRS‐R score	−0.17	0.29
ALSFRS‐R progression rate	0.17	0.29
MMSE	−0.13	0.59
PaCO_2_	*0.39	*0.03
PaO_2_	0.04	0.82
%VC	−0.14	0.44

*Note:* Spearman's correlation coefficient analysis was used to assess correlations.

Abbreviations: %VC, percentage vital capacity; ALSFRS‐R score, ALS Functional Rating Scale‐Revised score; MRC score, Medical Research Council sum score; PaCO_2_, partial pressure of carbon dioxide; PaO_2_, partial pressure of oxygen; *r*, correlation coefficient; UMN score: upper motor neuron score.

* A correlation was recognized between the p‐217tau level and PaCO_2_ (*r* = 0.39, *p* = 0.03).

### 3.4 Plasma p‐217tau and Clinical Phenotypes in ALS

The clinical phenotype (classical, flail arm, or PBP; Figure 2A); the affected sites at onset (bulbar, arms, or legs; Figure 2B); LMN and UMN signs in the bulbar, cervical, or lumbosacral region; and LMN signs in the thoracic region did not affect the plasma p‐217tau level (Data S1). Age at onset and the time between onset and blood sampling were not associated with plasma p‐217tau levels. There was no relationship between plasma p‐217tau levels and the UMN score, MRC sum score, ALSFRS‐R score, or the ALSFRS‐R progression rate. Plasma p‐217tau levels were weakly correlated with PaCO_2_ (*r* = 0.39, *p* = 0.03); however, they were not correlated with PaO_2_ or %VC (Table 3). In the EMG analysis, the existence of active denervation potentials in the bulbar region (*p* < 0.05) and chronic denervation in the cervical (*p* < 0.05), thoracic (*p* < 0.05), or lumbosacral (*p* = 0.06) region were found to be associated with higher plasma p‐217tau levels (Figure 2H,M–O). Thus, EMG findings of LMN lesions were associated with increased plasma p‐217tau levels.

### 3.5 Plasma p‐217tau Levels in iNPH

**FIGURE 3:**
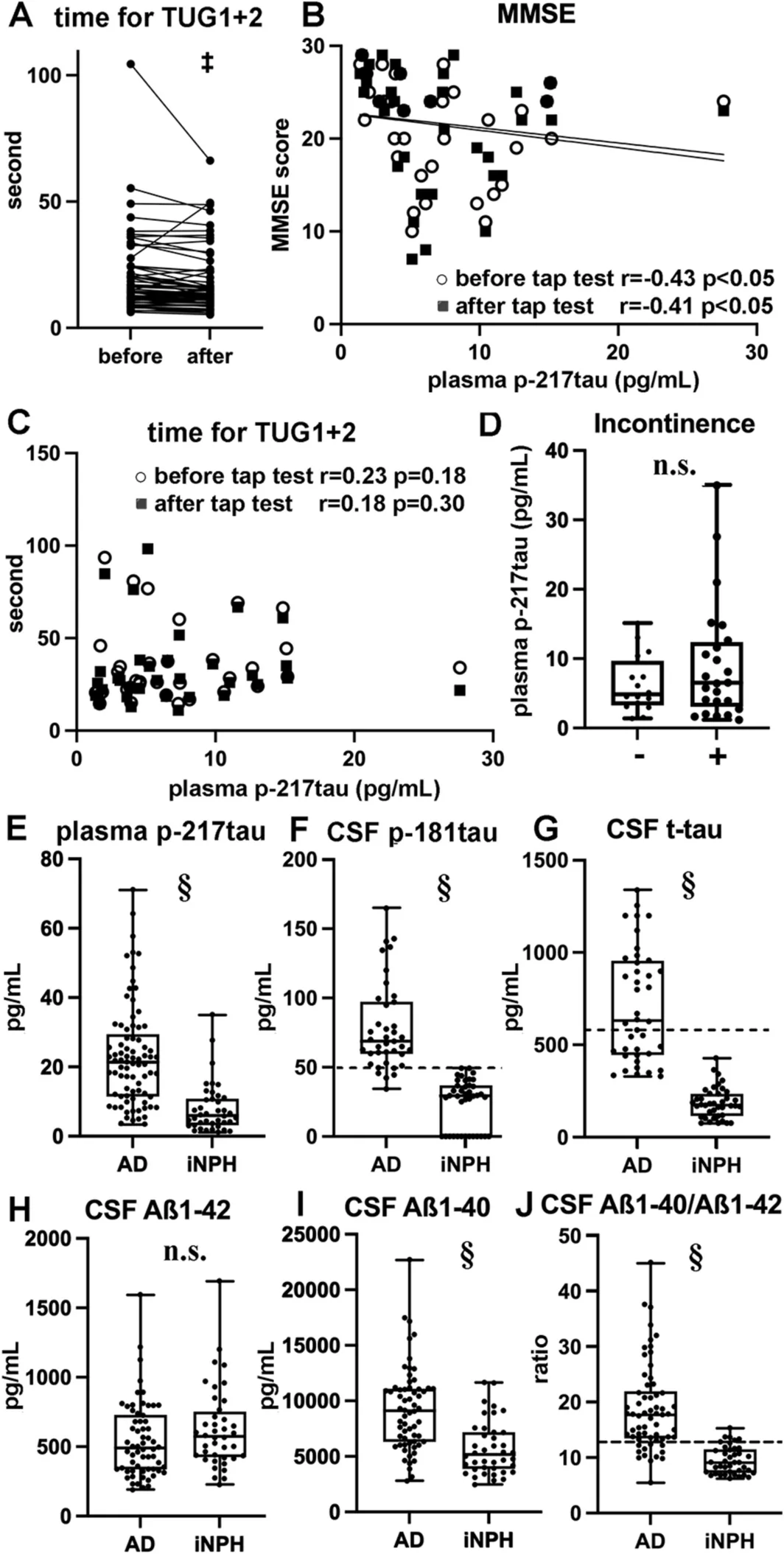
Plasma p‐217tau and clinical factors and other CSF biomarkers in iNPH. (A) The mean time for the TUG1 + 2 conducted before the tap test (19.0 ± 14.8 s) was significantly worse than those for the TUG1 + 2 conducted after the tap test (17.5 ± 12.0 s) (‡*p* < 0.001). (B) The plasma p‐217tau level and MMSE score were negatively correlated before (○ *r* = −0.43, *p* < 0.05; *Y* = −0.187 X + 22.8) and after (● *r* = −0.41, *p* < 0.05; *Y* = −0.165 X + 22.8) the tap test. (C) Gait disturbance, as assessed by the time for the TUG, was not correlated with the plasma p‐217tau level before (○*r* = 0.23, *p* = 0.18; *Y* = 0.258 X + 33.6) or after (● *r* = 0.18, *p* = 0.30; *Y* = −0.0339 X + 33.9) the tap test. (D) Plasma p‐217tau levels did not differ between the patients that were negative (6.35 ± 4.10 pg/mL) and positive (9.21 ± 8.56 pg/mL) for urinary incontinence (*p* = 0.47). Plasma p‐217tau (E), CSFp‐181tau (F), and CSF t‐tau (G) levels were significantly higher in the AD patients than in those with iNPH, being 2.8‐, 3.2‐, and 3.8‐fold higher, respectively (Table 2; §*p* < 0.0001). (H) CSF Aß1‐42 levels did not differ significantly between AD and iNPH (Table 2; *p* = 0.76). CSF Aß1‐40 (I) and the CSF Aß1‐40/Aß1‐42 ratio (J) were significantly lower in the iNPH patients than in the AD patients (Table 2; §*p* = 0.0001). ns, not significant; the dashed lines represent the threshold levels for the HCU.

The times for the TUG1 + TUG2 conducted after the tap test (17.54 ± 12.04 s) were significantly shorter than those for the TUG1 + TUG2 conducted before the tap test (18.98 ± 14.84 s, *p* < 0.001; Figure 3A). However, the MMSE scores obtained before and after the tap test did not differ significantly (*p* = 0.84). The MMSE scores obtained before and after the tap test were negatively associated with the plasma p‐217tau level before (*r* = −0.43, *p* < 0.05) and after (*r* = −0.41, *p* < 0.05; Figure 3B) the tap test. The MMSE score was also correlated with CSF t‐tau (*r* = −0.33, *p* < 0.05), Aß1‐42 (*r* = 0.38, *p* < 0.05), and Aß1‐40/Aß1‐42 levels (*r* = −0.37, *p* < 0.05) although these correlations were weaker than those for plasma p‐217tau (not shown). Gait disturbance, as assessed with the TUG time, was not correlated with the plasma p‐217tau level before (*r* = 0.23, *p* = 0.18) or after (*r* = 0.18, *p* = 0.30) the tap test (Figure 3C). Urinary incontinence was found in 60% of iNPH patients, and 17% of these patients exhibited improved urinary continence after the tap test. The plasma p‐217tau levels of the patients with and without urinary incontinence did not differ (*p* = 0.47; Figure 3D). Plasma p‐217tau, CSF p‐181tau, and CSF t‐tau levels were significantly higher in the AD patients than in the iNPH patients (2.8‐, 3.2‐, and 3.8‐fold higher, respectively) (*p* < 0.0001; Figure 3E–G). The CSF t‐tau levels of the iNPH patients were below the cut‐off value for AD (580.5 pg/mL, Figure 3G). The CSF Aß1‐42 level did not differ significantly between AD and iNPH (Figure 3H); however, CSF Aß1‐40 levels and the CSF Aß1‐40/Aß1‐42 ratio were significantly lower in iNPH than in AD (*p* = 0.0001; Figure 3I,J). The low levels of CSF t‐tau, p‐181tau, and CSF Aß1‐40 and the lower Aß1‐40/Aß1‐42 ratio suggested that the iNPH patients had fewer complications from AD pathology.

## 4 Discussion

In our AD group, plasma p‐217tau levels were markedly increased and rose with clinical progression. These results, which were obtained with the S‐PLEX assay in our Japanese cohort, correspond to those obtained in previous studies [17, 18, 37, 38]. ROC analysis of the utility of the plasma p‐217tau level for discriminating between AD patients and HCU showed that the AUC was 0.99, whereas the AUC for its utility for discriminating between AD and other neurological diseases was 0.93. Previous ROC analyses of CSF p‐217tau levels showed that the AUC for discriminating between AD patients and HCU was 0.98, whereas that for discriminating between AD and other neurological diseases was 0.95 [24]. Therefore, our data also indicate that the accuracy of the plasma p‐217tau assay used in this study is similar or superior to that of the previously reported CSF p‐217tau assay [15]. Among the 12 other neurological diseases examined in this study, CAA, DLB, FTD, CBS, PSP, PD, MSA, CCA, MS, and NMOSD did not show increased p‐217tau levels. It has already been reported that no increases in plasma p‐217tau levels were seen in PD, PD with dementia, MSA, PSP, FTD, or vascular dementia [37]. Except for Aß PET‐positive CBS‐ and AD‐related syndromes, including logopenic variant progressive aphasia and posterior cortical atrophy, plasma p‐217tau levels were not increased in CBS or progressive aphasia in previous studies [17, 18, 19]. Since Aß‐positive CBS and AD‐related syndromes are caused by AD pathology, these findings are reasonable. Piura showed that 33% of DLB patients, 22% of patients with vascular cognitive impairment (VCI), 12%–33% of patients with normal pressure hydrocephalus (NPH), 50% of patients with hippocampal sclerosis/limbic‐predominant age‐related TDP‐43 encephalopathy (LATE), and 12% of patients with other neurological diseases showed increased plasma p‐217tau levels [38]. These findings are interesting. Three of our CAA cases also showed high plasma p‐217tau levels. Among these conditions, comorbid AD pathology is a reasonable explanation for high plasma p‐217tau levels in DLB, VCI, and CAA. However, hippocampal sclerosis/LATE and the other neurological diseases are not associated with AD pathology. These findings imply that different mechanisms other than AD pathology may increase plasma p‐217tau levels. Since the plasma p‐217tau level is continuously increasing during the very long period from being cognitively unimpaired to the dementia stages of the AD continuum, careful evaluation to decide whether increases in plasma p‐217tau levels are caused by simple comorbid AD pathology or a different pathogenesis is critically important in the clinical setting and large‐scale clinical trials.

In our study, plasma p‐217tau levels were increased in ALS and the plasma p‐217tau levels of the ALS patients were between those seen in the AD patients and HCU, as reported previously [20, 39]. The CSF p‐217tau, p‐181tau, and t‐tau levels of the ALS patients were slightly elevated, as reported previously [24], but the increases were not significant. The plasma p‐217tau/CSF p‐217tau ratio was inversely higher in ALS than in AD. Plasma p‐217tau levels were strongly correlated with CSF p‐217tau levels in AD; however, they were not correlated in ALS. No correlations were observed between plasma p‐217tau and CSF p‐181tau levels or between plasma p‐217tau and CSF t‐tau levels in ALS. In AD, plasma p‐217tau is derived from the brain and CSF, from which it moves into plasma, as has been shown in a human kinetic study [40], suggesting that plasma p‐217tau may have a different origin in ALS. The clinical phenotype of ALS, the affected sites, clinical LMN and UMN signs, age at onset, time to blood sampling, UMN score, MRC sum score, ALSFRS‐R score, and ALSFRS‐R progression rate were not correlated with the plasma p‐217tau level. Plasma p‐217tau levels were not correlated with PaO_2_ or %VC; however, they were weakly correlated with PaCO_2_. On the contrary, active or chronic denervation potentials in the bulbar, cervical, thoracic, or lumbosacral regions were associated with high plasma p‐217tau levels. One study has reported that short duration from the onset and high ALSFRS‐R scores in spinal onset ALS showed high p‐217tau levels [41]. Another study showed that there was no relationship among serum p‐217tau levels, the time since onset, and the ALSFRS‐R score [20]. Our EMG study showed that active and chronic denervation both increase plasma p‐217tau levels, suggesting that LMN disturbances are associated with plasma p‐217tau level increases. Cousins was the first to report that high plasma p‐181tau levels are associated with LMN clinical signs and neuronal loss in the spinal cord, but not in the motor cortex in ALS [42]. EMG studies showed that high plasma p‐181tau levels are correlated with denervation findings [33, 43]. In addition, high plasma p‐217tau and p‐181tau levels were suggested to be associated with LMN lesions. Recent studies proposed two possibilities: One is the disturbed muscle origin hypothesis involving Big tau phosphorylated at Thr217 (Big p‐217tau) [20, 44]. The second hypothesis involves increases in Big p‐217tau levels derived from peripheral nerve systems [39]. Recently, a low molecular weight (LMW) brain‐specific p‐217tau assay that is able to discriminate between AD and ALS with greater precision was developed [39]. However, higher expression levels of Big tau have been found in the visual system, cerebellum, brainstem, spinal motor neurons, dorsal root ganglions, and peripheral nerves [44, 45]. The significant overlap in plasma assay levels between LMW p‐217tau and Big p‐217tau levels found in controls and ALS and AD patients suggests the possibility that both LMW tau and Big tau comigrate into plasma [39]. Since S‐PLEX has not been shown to selectively detect LMW p‐217tau, the elevated plasma p‐217tau levels seen in ALS suggest that the S‐PLEX assay may not be able to differentiate between Big p‐217tau and LMW brain p‐217tau. Further elucidation of precise LMW p‐217tau and Big p‐217tau ratios and an efflux kinetic study [40] of these molecules are necessary to address the origin of plasma p‐217tau in ALS.

iNPH is another disorder that showed high plasma p‐217tau levels. The plasma p‐217tau levels of the iNPH patients were almost the same as those seen in ALS. Although the comorbidity rate of AD in iNPH is high, recent studies examining brain biopsy pathology and amyloid PET images showed that 43%–66% of iNPH cases were not associated with AD, indicating that not all iNPH cases are caused by underlying AD pathology. It has been reported that concomitant AD pathology causes poor outcomes in shunt surgery [46, 47, 48, 49, 50, 51]. For this reason, it is necessary to check patients' AD biomarker levels to estimate the likelihood of them having comorbid AD when deciding on the indications for shunt surgery and predicting prognoses. In the Kuopio NPH registry, cases of iNPH without AD pathology (according to brain biopsies) did not show increased CSF levels of t‐tau or p‐181tau; however, they did exhibit moderately decreased Aß levels [52]. For this reason, we strictly selected iNPH cases based on the Guidelines for Management of Idiopathic Normal Pressure Hydrocephalus (Third Edition), which require the presence of DESH, an improvement in the TUG result after CSF drainage, and low CSF t‐tau and p‐181tau levels, and a low Aß40/42 ratio. Since our iNPH subjects did not show the AD biomarker profile, the likelihood of Aß or tau accumulation occurring was low. However, the plasma p‐217tau level was increased in our iNPH subjects, despite no other AD CSF biomarkers exhibiting increased levels. This is the reason why we speculate that a different mechanism is responsible for the increased plasma p‐217tau levels seen in iNPH. Actually, plasma p‐217tau is a core 1 biomarker of AD with extremely high sensitivity and specificity. If plasma p‐217tau was used for mass screening for AD, cases of iNPH involving increased plasma p‐217tau levels, but no increases in the levels of other AD CSF biomarkers, would be misdiagnosed. For this reason, we insist that iNPH is a pitfall for the clinical application of plasma p‐217tau as a biomarker of AD.

The plasma p‐217tau level was negatively correlated with the MMSE score; however, no association with gait or incontinence was recognized. Thus, the plasma p‐217tau level is associated with cognitive impairment in iNPH. Although iNPH is assumed to be caused by impaired CSF dynamics, the redistribution of intracranial pulsatility from the subarachnoid space to the ventricle, reductions in cerebral blood flow, impaired glymphatic clearance, reduced blood–brain barrier integrity, and alterations in venous hemodynamics were hypothesized to be involved [53]. Recently, significant improvement was reported in a randomized trial of shunting for iNPH [54]. Thus, the indications for shunt surgery should be carefully considered, even in iNPH patients with high plasma p‐217tau levels. Further study of p‐217tau kinetics in CSF and plasma may also be necessary to elucidate the mechanism for increasing plasma p‐217tau levels in iNPH, which is different from that in AD pathology or the LMN degeneration in ALS.

The main limitations of this study were the fact that it only included a small number of subjects, especially HCU. We selected control subjects without any systemic or neurological complications. This approach reduced the number and mean age of the HCU. The next limitation is that age and sex were not matched to the HCU in some diseases. As sex‐related differences in plasma p‐217tau levels have been reported, this may have affected our results. The third limitation is that CSF biomarkers were not measured in all AD, ALS, and iNPH patients. The fourth limitation is that amyloid PET was not performed. Therefore, complicating AD pathology was not fully eliminated in ALS or iNPH. The fifth limitation is that we did not measure LMW brain p‐217tau and Big p‐217tau levels separately. If we had done this, we could have examined the origins of plasma p‐217tau in AD, ALS, and iNPH more precisely.

Our study showed that plasma p‐217tau is an excellent biomarker for identifying AD pathology; however, ALS and iNPH are two major pitfalls for the clinical application of plasma p‐217tau as a specific biomarker of AD. LMN injuries in ALS and cognitive dysfunction in iNPH are both contributing factors for increased plasma p‐217tau levels. In clinical evaluations of plasma p‐217tau levels, it is important to be aware that plasma p‐217tau levels may be increased by mechanisms unrelated to AD pathology.

## Author Contributions

T.K. M.S., and T.U. contributed to the conception and design of the study; T.K., T.N., R.T., S.K., C.U., T.S., K.H., K.I., M.A., H.K., Y.I., and M.T., and M.S., T.S., K.I., M.A., R.T., H.K., T.K., and Y.I. contributed to the acquisition and analysis of the data, respectively; T.K., T.U., and M.S. contributed to drafting a significant portion of the manuscript or figures.

## Funding

This work was supported by JSPS KAKENHI Grant‐in‐Aid for Scientific Research (C) from the Ministry of Education, Science, and Culture of Japan, 18K07385, 22K07511.

## Conflicts of Interest

The authors declare no conflicts of interest.

## Supporting information


**Data S1:** (A–D) Changes in plasma p‐217tau levels according to the presence/absence of lower motor neuron signs (muscle atrophy and hypotonia or fasciculation) in the bulbar (negative: 6.65 ± 4.26 pg/mL, *n* = 6; positive: 6.93 ± 4.33 pg/mL, *n* = 24; *p* = 0.73), cervical (negative: 4.65 pg/mL, *n* = 1; positive: 6.87 ± 4.29 pg/mL, *n* = 39), thoracic (negative: 6.42 ± 3.51 pg/mL, *n* = 10; positive: 6.95 ± 4.51 pg/mL, *n* = 30; *p* = 0.94), or lumbosacral (negative: 5.18 ± 2.57 pg/mL, *n* = 9; positive: 7.29 ± 4.55 pg/mL, *n* = 31; *p* = 0.17) region. (E–G) Changes in plasma p‐217tau levels according to the presence/absence of upper motor neuron signs (hyperreflexia, spasticity, or pathological reflexes) in the bulbar (negative: 6.92 ± 5.67 pg/mL, *n* = 13; positive: 6.77 ± 3.5 pg/mL, *n* = 27; *p* = 0.32), cervical (negative 6.79 ± 4.97 pg/mL, *n* = 10; positive: 6.83 ± 4.08 pg/mL, *n* = 30; *p* = 0.82), or lumbosacral (negative: 7.02 ± 6.56 pg/mL, *n* = 6; positive: 6.78 ± 3.85 pg/mL, *n* = 34; *p* = 0.62) region. None of these parameters affected plasma p‐217tau levels. +, positive; −, negative; ns, not significant.

## Data Availability

Data sharing is available on request.
