# Supplementary material for: Clinical Validation of Plasma p‐217tau in Neurological Diseases
Source: Ann Clin Transl Neurol. 2026 Mar 9;13(9):1780–90. doi: 10.1002/acn3.70359 (PMC13394761; doi:10.1002/acn3.70359)
Supplement: Supplementary file 1 [file ACN3-13-1780-s001.docx]

Supplementary Data

(A-D) Changes in plasma p-217tau levels according to the presence/absence of lower motor neuron signs (muscle atrophy and hypotonia, or fasciculation) in the bulbar (negative: 6.65±4.26 pg/mL, n=6; positive: 6.93±4.33 pg/mL, n=24; p=0.73), cervical (negative: 4.65 pg/mL, n=1; positive: 6.87±4.29 pg/mL, n=39), thoracic (negative: 6.42±3.51 pg/mL, n=10; positive: 6.95±4.51 pg/mL, n=30; p=0.94), or lumbosacral (negative: 5.18±2.57 pg/mL, n=9; positive: 7.29±4.55 pg/mL, n=31; p=0.17) region. (E-G) Changes in plasma p-217tau levels according to the presence/absence of upper motor neuron signs (hyperreflexia, spasticity, or pathological reflexes) in the bulbar (negative: 6.92±5.67 pg/mL, n=13; positive: 6.77±3.5 pg/mL, n=27; p=0.32), cervical (negative 6.79±4.97 pg/mL, n=10; positive: 6.83±4.08 pg/mL, n=30; p=0.82), or lumbosacral (negative: 7.02±6.56 pg/mL, n=6; positive: 6.78±3.85 pg/mL, n=34; p=0.62) region. None of these parameters affected plasma p-217tau levels. n.s.: not significant, +: positive, -: negative
